# Genetic Variant in Interleukin-18 Is Associated with Idiopathic Recurrent Miscarriage in Chinese Han Population

**DOI:** 10.3390/ijms16024180

**Published:** 2015-02-16

**Authors:** Jun Yue, Yu Tong, Jing Zhou, Qingqing Liu, Jiyun Yang

**Affiliations:** 1 Department of Gynecology & Obstetrics, Hospital of the University of Electronic Science and Technology of China and Sichuan Provincial People’s Hospital, No. 32, Section 2, the Western First Round Road, Chengdu 610072, China; E-Mail: yuejunlinda@sina.com; 2 Laboratory of Early Developmental and Injuries, West China Institute of Woman and Children’s Health, West China Second University Hospital, Sichuan University, No. 20, Section 3, Renmin Nanlu, Chengdu 610072, China; E-Mails: toyuer@gmail.com (Y.T.); Liuqingqing@163.com (Q.L.); 3 Key Laboratory of Gynecologic & Obstetric and Pediatric Diseases and Birth Defects of Ministry of Education, Chengdu 610072, China; 4 Department of Laboratory Medicines, West China Hospital, Sichuan University, No. 37, Guo Xue Xiang, Chengdu 610072, China; E-Mail: zhoujing@163.com; 5 Sichuan Provincial Key Laboratory for Human Disease Gene Study, Hospital of the University of Electronic Science and Technology of China and Sichuan Provincial People’s Hospital, No. 32, Section 2, the Western First Round Road, Chengdu 610072, China; 6 School of Medicine, University of Electronic Science and Technology of China, No. 32, Section 2, the Western First Round Road, Chengdu 610072, China

**Keywords:** idiopathic recurrent miscarriage, interleukin-18, polymorphism

## Abstract

Levels of IL-18 were significantly lower in women with recurrent miscarriage (RM) than those without idiopathic RM. IL-18 promoter single nucleotide polymorphisms were previously identified to have an impact on IL18 gene transcription activity and influence the level of IL-18 protein production. The aim of this study was to evaluate whether IL-18 gene polymorphisms are risk factors for idiopathic RM in Chinese Han population. Study subjects comprised of 484 idiopathic RM patients and 468 controls. Three polymorphisms (rs360717, rs187238, rs1946518) in IL-18 gene and serum IL-18 concentrations were assessed. rs187238 variant exhibits significant association with RM in additive and recessive genetic model (additive model *p* = 1.05 × 10^−4^, dominant model *p* = 0.025, recessive model *p* = 2.43 × 10^−5^). In contrast, rs360717 and rs1946518 are not significantly associated with RM. Serum IL-18 levels are significantly lower in RM cases than in control (111.98 ± 93.13 *versus* 148.74 ± 130.51 pg/mL, *p* = 7.42 × 10^−7^). There are lower levels of serum IL-18 in rs187238 homozygous mutant (CC) than homozygous wild-type (GG) in this study population, including cases and control groups (98.31 ± 86.46 *versus* 131.87 ± 115.02 pg/mL, *p* = 0.015). These results suggest that reduced IL-18 levels and rs187238 variant may contribute to pathogenesis of idiopathic RM in Chinese Han population.

## 1. Introduction

Recurrent miscarriage (RM) is the occurrence of three or more repeated pregnancies that end in loss of the fetus, usually before 20 weeks of gestation. RM affects 1%–5% of women who attempting to bear children [1]. A series of etiological factors have been identified for miscarriage, including uterine anomaly, chromosomal abnormalities, endocrine dysfunction, thrombophilia, immune disorders, life style factors and maternal infections [1]. However, At least 50% of the RM patients have no deviations in any applied diagnostic test. The exact underlying etiology is still poorly understood. There is growing evidence that RM has also genetic susceptibility [2,3,4].

Production of cytokines and the distribution of the immune cells during pregnancy appeared to be critical in successful pregnancy [5,6,7]. As a member of the IL-1 family of cytokines, interleukin-18 (IL-18) is produced by a variety of immune and non-immune cells, including endothelial cells, keratinocytes, intestinal epithelial cells, macrophages and dendritic cells [8,9,10]. As a pleiotropic proinflammatory cytokine, IL-18 is actively involved in the regulation of immune responses and enhances either Th1 or Th2 differentiation depending on the immunologic milieu [11]. Many studies have explored the role of IL-18 in mediating inflammation, it is involved in pathogenesis of several autoimmune diseases, myocardial function, emphysema, metabolic syndromes, psoriasis, inflammatory bowel disease, macrophage activation syndrome, hemophagocytic syndromes, acute kidney injury and sepsis [12,13,14].

A few studies have showed that polymorphisms of IL-18 gene may be involved in the association of recurrent miscarriage [15,16,17]. Three SNPs (rs360717, rs187238, rs1946518) in the promoter region of IL-18 gene were identified to have an impact on IL18 gene transcription activity and influence the level of IL-18 protein production [18,19]. Recent report indicates that reduced IL-18 levels and rs360717 and rs1946519 IL-18 variants are significantly associated with RM in Bahraini and Tunisian population [16,20]. It remains unknown whether genetic variant in the promoter region of IL-18 gene is associated with idiopathic recurrent miscarriage in Chinese Han population.

Here, we conducted a case-control study to explore the association between polymorphisms in the promoter region of IL-18 gene and idiopathic RM in Chinese Han population. Three polymorphisms (rs360717, rs187238, rs1946518) in the promoter region of IL-18 gene were selected for genotyping, and then we analyzed the association between the three SNPs and the risk of idiopathic recurrent miscarriage. 

## 2. Results and Discussion

### 2.1. Results

#### 2.1.1. The Clinical Characteristics of Study Participants

The clinical characteristics of patients and controls are listed in Table 1. There are no significant differences in mean age, smoking (%) and mean BMI (kg/m^2^) between cases and control (*p >* 0.05). While lower age at menarche, higher irregular menstrual history (%) and number of pregnancies were seen in the RM group. Although they did not constitute strong risk factors of RM, they were selected as the covariates that were controlled for in subsequent analyses. 

**Table 1:** Clinical characteristics of the study population.

Characteristic	Cases	Controls	*p* Value
Mean age (years)	31.40 ± 5.08	31.97 ± 4.39	0.061
Smoking (%)	36 (7.44)	45 (9.61)	0.246
Mean BMI (kg/m^2^)	20.89 ± 3.00	21.22 ± 2.89	0.079
Menarche (years)	13.11 ± 1.16	13.30 ± 1.43	0.025
Irregular menstrual history (%)	63 (13.02)	38 (8.12)	0.015
Number of pregnancies	4.89 ± 1.19	2.98 ± 1.27	<0.0001
Abortion	3.82 ± 0.731	0 ± 0	<0.0001

#### 2.1.2. IL-18 Genotype Distribution

Genotype distribution of three (rs360717, rs187238 and rs1946518) SNPs are in Hardy-Weinberg equilibrium in cases and controls (*p* > 0.05). 

Table 2 summarizes the distribution of IL-18 genotypes between patients and control. Significant differences in the distribution of rs187238 genotype are observed between patients and controls. rs187238 variant exhibits significant association with RM in any of three genetic models (additive model *p* = 1.05 × 10^−4^, dominant model *p* = 0.025, recessive model *p* = 2.43 × 10^−5^). As shown in Table 3, after adjustments for menarche age, irregular menstrual history and number of pregnancies by Binary Logistic Regression, this association remains significant in two genetic models (adjusted odds ratio (OR) = 4.346, 95% confidence interval (CI) = 1.257–15.029, *p* = 0.021 in an additive model; OR = 3.732, 95% CI = 1.132–12.309, *p* = 0.037 in a recessive model). In contrast, rs360717 and rs1946518 are not significantly associated with RM in any of three genetic models. 

**Table 2:** Association between three SNPs in the promoter region of IL-18 gene and risk for idiopathic recurrent miscarriage in Chinese Han population.

SNP	Genotype	Case (*n* = 484)	Control (*n* = 468)	Additive *p* Value	Dominant *p* Value	Recessive *p* Value
rs360717	GG	349 (0.721)	332 (0.709)	0.800	0.690	0.547
	AG	129 (0.267)	128 (0.274)			
	AA	6 (0.012)	8 (0.017)			
rs187238	GG	338 (0.758)	357 (0.763)	1.05 × 10^−4^	0.025	2.43 × 10^−5^
	GC	108 (0.223)	102 (0.218)			
	CC	38 (0.079)	9 (0.019)			
rs1946518	AA	181 (0.374)	178 (0.380)	0.905	0.839	0.656
	AC	216 (0.446)	211 (0.451)			
	CC	87 (0.180)	79 (0.169)			

**Table 3:** Associated between rs187238 variant with risk for idiopathic recurrent miscarriage after adjustment for Binary Logistic Regression.

Model	*p* Value	Wald Value	OR	95% CI
Additive	0.021	5.345	4.343	1.250–15.009
Dominant	0.298	1.084	1.245	0.824–1.881
Recessive	0.015	5.909	4.343	1.329–14.190

OR, odds ratio; CI, confidence interval.

#### 2.1.3. Mean Serum IL-18 Levels

**Figure 1:**
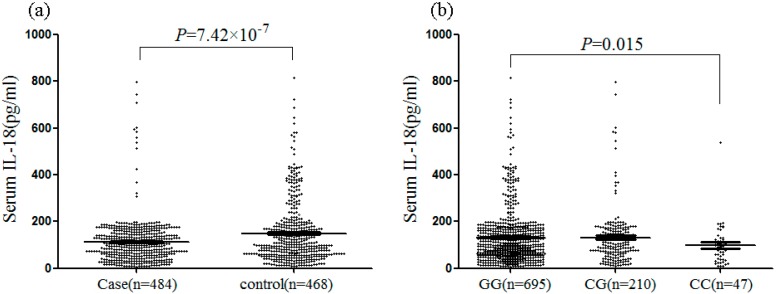
Comparison of serum IL-18 levels among IL18 genotypes. Serum IL-18 was measured using a human IL-18 enzyme-linked immunosorbent assay. Data were expressed as means ± SD. Statistical differences were performed using Mann-Whitney U test for variance non-homogeneity. (**a**) Serum IL-18 concentrations in cases and controls; (**b**) Serum IL-18 levels according to rs187238 genotypes among cases and controls.

As shown in Figure 1A, serum IL-18 levels are significantly lower in RM cases than in control (111.98 ± 93.13 *versus* 148.74 ± 130.51 pg/mL, *p* = 7.42 × 10^−7^). As shown in Figure 1A, there are lower levels of serum IL-18 in rs187238 homozygous mutant (CC) than homozygous wild-type (GG) in this study population, including cases and control groups (CC *versus* GG, 98.31 ± 86.46 *versus* 131.87 ± 115.02 pg/mL, *p* = 0.015. Moreover, there is a significant decline in serum IL-33 levels according to rs187238 genotypes in RM cohorts (CC *versus* GG, 106.46 ± 75.69 *versus* 131.77 ± 132.85 pg/mL, *p* = 0.014). However, there is no significantly difference between the group with homozygous mutant (CC) and the group (GG + CG) with G allele (CC *versus* GG + CG 98.31 ± 86.46 *versus* 121.69 ± 117.60 pg/mL, *p* = 0.072). 

### 2.2. Discussion

Idiopathic RM is characterized by multifactorial etiology. Although much work has been done to identify the cause of idiopathic RM, its reason remains unknown [1]. There is growing evidence that RM has also genetic susceptibility. In most conducted genetic association studies targeting RM, the most frequently addressed genes in the context of RM are associated with immunotolerance, inflammation and changes of maternal metabolism and blood coagulation [2]. 

A few studies have shown that polymorphisms of IL-18 gene may be involved in the association of recurrent miscarriage [15,16,17]. Specifically, variants in the promoter region of IL-18 gene influence the level of IL-18 protein production [18,19]. IL-18, originally identified as an IFN-γ-inducing factor, is a pro-inflammatory cytokine with a unique capacity to induce T helper 1 or T helper 2 cells polarization depending on the immunologic context [21]. The high production of IL-18 has been implicated in the pathogenesis of several immune-mediated processes [22]. Successful pregnancy depends on the cytokine environment, which can either be protective or harmful to the conceptus [23]. Levels of IL-18 were significantly lower in women with RM than those without RM [24]. Research also shows that the production of IL-18 is mediated by cleavage of caspase-1. Expression of both caspase 1 and IL-18 are essential for pregnancy stability in pigs [25].

A few studies have been reported on the association of IL-18 variants with RM but with inconclusive findings. −105G/A (rs360717) and −656C/A (rs1946519) IL-18 variants are significantly associated with RM in 470 Tunisian women comprising 235 RM cases and 235 multi-parous controls. For −137G/C (rs187238), −119A/C (rs360718), no significant association with RM was found in this study [20].

This was in agreement with a case-control study in Arabic population from Bahraini [16]. Both rs360717 and rs1946519 IL-18 single-nucleotide polymorphisms, but not rs360718 and rs187238, showed significant association with RM under additive, dominant, and recessive models. Lower serum IL-18 levels were seen between patients and controls and were more pronounced in rs360717 and rs1946519 heterozygous and homozygous genotypes [16].

Meanwhile, in three ethnic populations with RM, including southern Iranian, Arabic population from Bahraini and Slovenian women, IL-18 gene promoter polymorphisms at positions −607 (rs1946518) and −137 (rs187238) did not confer susceptibility to RM [15,16,17]. Additional studies on larger numbers of women with RSM from different ethnic backgrounds are needed to confirm the association of IL-18 gene promoter polymorphisms with RM.

In this study, significant differences in the distribution of rs187238 genotype are observed between patients and controls from Chinese Han population. rs187238 variant exhibits significant association with RM in any of three genetic models. After adjustments for menarche age, irregular menstrual history and number of pregnancies by Binary Logistic Regression, this association remains significant in two genetic models (adjusted odds ratio (OR) = 4.346, 95% confidence interval (CI) = 1.257–15.029, *p* = 0.021 in an additive model; OR = 3.732, 95% CI = 1.132–12.309, *p* = 0.037 in a recessive model). However, in apparent disagreement with those study [15,16,17,20], rs360718 and rs360717 are not significantly associated with RM in any of three genetic models in this study. The minor allele frequencies of three variants among control women from Chinese Han population were different to frequencies for white individuals, African Americans, or sub-Saharan Africans. These findings confirm racial heterogeneity in the distribution of IL-18 variants and hence their disease association.

IL-18 promoter single nucleotide polymorphisms previously implicated in altered IL-18 secretion. We found that serum IL-18 levels are significantly lower in idiopathic RM cases than in control, in agreement with previous findings documenting reduced IL-18 levels in patients with confirmed repeated miscarriage [6,16,24]. This indicates that optimal IL-18 levels are critical for successful pregnancy. In this study, there are lower levels of serum IL-18 in rs187238 homozygous mutant (CC) than homozygous wild-type (GG) in this study population, including cases and control groups. This may be linked with influencing the transcriptional rate and therefore gene expression of IL-18.

In conclusion, our study demonstrated that the rs187238 variant in IL-18 promoter, but not variants rs360718 and rs360717, is associated with RM. Our study has strengths, namely that it was sufficiently powered, that cases and controls were ethnically matched, hence minimizing the problems of differences in genetic background. However, our study has some limitations. It was limited to Chinese Han population, thereby necessitating parallel studies on different ethnic groups. Follow-up studies on additional IL-18 variants, and populations of related and distant ethnic origins are needed to confirm the association of IL-18 variants with altered IL-18 secretion and risk of RM. Functional experiments are also needed to elucidate the association.

## 3. Experimental Section 

### 3.1. Patients and Controls

This study has been carried out in accordance with The Code of Ethics of the World Medical Association (Declaration of Helsinki) for experiments involving humans. The Institutional Review Boards of the Sichuan Provincial People’s Hospital and West China Second University Hospital, Sichuan University approved this study. All subjects provided informed consent before participating in the study.

Cases comprised 484 women from Sichuan Provincial People’s Hospital and West China Second University Hospital between January 2011 and July 2014, who had experienced three or more pregnancy losses with the same partner, and which had occurred from between the beginning of pregnancy to the 20th week of gestation; gestational age was calculated as the time between the first day of the last normal menstrual period and the first signs of pregnancy loss. The work-up included endometrial biopsies for evaluating luteal phase defects; pelvic ultrasound scans to assess ovarian morphology and the uterine cavity; hysteroscopy to evaluate uterine anatomic abnormalities; karyotyping of peripheral blood to evaluate chromosomal aberrations in of both patient and partner. These procedures were performed on all patients.

Exclusion criteria included older age (40 years or older at first miscarriage), Rh incompatibility, anatomical abnormalities, preclinical miscarriages, endocrine disorders (including diabetes), liver function abnormalities, and abnormal thyroid function, thyroid antibodies, hyperprolactinemia prior to luteal phase defects, fetomaternal alloimmune thrombocytopenia, infections (toxoplasmosis, human cytomegalovirus, rubella, human immunodeficiency virus, Group B streptococci, Chlamydia trachomatis, hepatitis B and C and bacterial vaginosis), parental and fetal chromosomal aberrations. Trisomy and triploidy of fetal tissues were evaluated using fluorescence *in situ* hybridization (FISH). Data on FISH of fetal tissues were available for 421 (86.98%) patients. Patients were also excluded if they reported systemic autoimmune disease, such as anti-phospholipid syndrome, systemic lupus erythematosus, multiple sclerosis, rheumatoid arthritis.

Control subjects composed of 468 women who were recruited from Sichuan Provincial People’s Hospital and West China Second University Hospital between January 2011 and July 2014. Age and ethnical group were matched with patients. All subjects had at least one live birth and no miscarriages, and did not have a family history of RM. 

### 3.2. IL-18 Genotyping

Blood samples were taken from all participants in EDTA containing tube for total genomic DNA extraction. Total genomic DNA was extracted by silica columns isolation kits (Tiangen BioTech Co., Ltd., Beijing, China).

Genotype information of a total of five polymorphisms in the promoter region of IL-18 gene was derived from the HapMap CHB populations (http://hapmap.ncbi.nlm.nih.gov/cgi-perl/gbrowse/hapmap27_B36/). Genotype information of rs187238 variant was not found in HapMap CHB populations. rs360717 and rs360718, rs1946518 and rs1946519 were located in the same block with r-squared of 1. Three SNPs (rs360717, rs187238, rs1946518) in the promoter region of IL-18 gene were identified to have an impact on IL18 gene transcription activity and influence the level of IL-18 protein production. So, we finally selected polymorphism rs360717, rs187238 and rs1946518 for association studies.

IL-18 genotyping was performed using real time PCR assays (TaqMan probe). TaqMan^®^ SNP Genotyping Assay kit were from Applied Biosystems (Carlsbad, CA, USA). Cat. C_2898462_10 (rs360717), C_2408543_10 (rs187238), C_2898460_10 (rs1946518). Replicate blinded quality control samples were included to assess the reproducibility of the genotyping procedure. The successful genotyping rate was 99.0%. In brief, 48 individuals of cases and controls were randomly selected to sequence for confirmation of three SNPs genotyping. The confirmation rate was 100%.

### 3.3. Serum IL-18 Measurements

Because pregnancy influences IL-18 levels, blood drawing criteria were used for patients and control women. This included interval (4–6 months) from the last event (miscarriage or delivery) and time of blood draw (8:00–10:00 a.m.). Blood samples were taken from all participants in plain tubes (no preservatives) for serum preparation. Serum was prepared by centrifugation of coagulated blood tubes at 2000× *g* for 10 min at room temperature, and was stored in 200 μL aliquots at −80 °C. Serum IL-18 was measured using a human IL-18 enzyme-linked immunosorbent assay (catalogue number 7620; MBL International Corporation, Woburn, MA, USA). According to the manufacturer’s instructions. Assay sensitivity was 12.5 pg/mL. Samples were tested in triplicate at the same time in the same assay.

### 3.4. Statistical Analysis

Statistical analysis was performed by the software SPSS for Windows (version 13.0) (SPSS Inc., Chicago, IL, USA). Continuous variables were expressed as mean ± SD. Statistical differences were performed using *t* test or Mann–Whitney U test. Then differences in genotype distribution of the polymorphism between case and control subjects were compared by χ^2^ test. Three models were used for statistical analysis, including dominant model, the recessive genetic model and the additive model. Logistic regression analysis was used to evaluate the association between genotype of SNPs and idiopathic recurrent miscarriage. *p* value of less than 0.05 was considered statistically significant. Gene variants were tested for Hardy-Weinberg equilibrium using Haploview version 4.2 (http://www.broad.mit.edu/mpg/haploview).

We used CaTS Power Calculator (www.sph.umich.edu/csg/abecasis/cats) to calculate the power to detect an association between IL-18 variants and RPL in our cohort. The parameters used were: 484 RM cases and 468 control women, genotypic relative risk for heterozygotes (1/2) and minor allele homozygotes (2/2), and the MAF for RM cases and controls for the five tested SNPs, and assumed 5% population prevalence of RM. Assuming these parameters, this sample size provided 84%, 88% and 99% power for rs360717, rs187238 and rs1946518 respectively.

## 4. Conclusions

In conclusion, our study found that the rs187238 variant in IL-18 promoter was associated with idiopathic RM, and the CC genotype of rs187238 is associated with lower serum IL-18 levels. These results suggest that reduced IL-18 levels and rs187238 variant may contribute to pathogenesis of idiopathic RM in Chinese Han population.
